# Management of chronic unstable acromioclavicular joint injuries

**DOI:** 10.1007/s10195-017-0452-0

**Published:** 2017-03-08

**Authors:** Luis Natera Cisneros, Juan Sarasquete Reiriz

**Affiliations:** 1 Hospital de la Santa Creu i Sant Pau, Universitat Autònoma de Barcelona, Street Sant Quintí 89, 08026 Barcelona, Spain; 2 Hospital General de Catalunya, Street Pedro i Pons 1, 08190 Sant Cugat Del Vallés, Barcelona Spain; 3 Hospital Quirón Teknon, Street Vilana 12, 08022 Barcelona, Spain

**Keywords:** Unstable acromioclavicular joint injuries, Chronic setting, Arthroscopically assisted management, Anatomical ligament reconstruction, Coracoclavicular ligaments, Scapular dyskinesis

## Abstract

****Abstract
**:**

The acromioclavicular joint represents the link between the clavicle and the scapula, which is responsible for the synchronized dynamic of the shoulder girdle. Chronic acromioclavicular joint instability involves changes in the orientation of the scapula, which provokes cinematic alterations that might result in chronic pain. Several surgical strategies for the management of patients with chronic and symptomatic acromioclavicular joint instability have been described. The range of possibilities includes anatomical and non-anatomical techniques, open and arthroscopy-assisted procedures, and biological and synthetic grafts. Surgical management of chronic acromioclavicular joint instability should involve the reconstruction of the torn ligaments because it is accepted that from three weeks after the injury, these structures may lack healing potential. Here, we provide a review of the literature regarding the management of chronic acromioclavicular joint instability.

**Level of evidence:**

Expert opinion, Level V.

## Introduction

The acromioclavicular joint (ACJ) represents the link between the clavicle and the scapula, which is responsible for the synchronized dynamic of the shoulder girdle [1]. It has been shown that most patients with a history of unstable ACJ injuries managed conservatively develop changes in the anatomical orientation of the scapula, which provokes alterations in the dynamics of the rotator cuff, which can eventually predispose chronic pain [2].

Biomechanical studies have demonstrated the importance of anatomical reconstruction of the coracoclavicular (CC) ligaments in cases of unstable ACJ injuries [3]. This importance lies in the fact that the conoid and trapezoid ligaments have different functions, which depend on their anatomical location and orientation [4].

Many of the procedures for the treatment of unstable ACJ injuries are non-anatomical [5]. The therapeutic approach for chronic ACJ instability should be different from that for acute ACJ instability. In the acute phase, it is accepted that the acromioclavicular (AC) and CC ligaments still have the potential to heal, so surgical techniques may aim to align the ends of the torn ligaments while tissue-healing takes place [6]. On the other hand, as the AC and CC ligaments lose their potential to heal from 3 weeks after the ACJ injury [6], the management of chronic ACJ instability must involve biological augmentation as well as mechanical fixation [7].

Many strategies that have been described for the management of chronic ACJ instability are non-anatomical [8] and lack primary mechanical fixation [9] that protects the graft during integration to the bone.

Here, we present a review of the literature regarding the management of chronic unstable ACJ injuries. As this review is narrative, we only included studies that were found to be of interest in supporting the concepts that we aim to transmit.

## Surgical management

### Indications for treatment

It is currently accepted that reasonable management for grade III ACJ injuries consists of conservative measures. A second examination (3–6 weeks after shoulder injury) must be carried out to assess the evolution of symptoms. If at 3 months after the shoulder injury (already in chronic phase) a patient with a grade III ACJ injury still complains of symptoms of scapular dyskinesis, and radiographic examinations show overriding of the distal third of the clavicle over the acromion in the Alexander projection, surgical treatment is recommended [10].

Patients with chronic and symptomatic ACJ instability (Rockwood grade III–V) must be informed about the internationally accepted recommendations regarding the surgical treatment of these injuries once the conservative measures have failed. However, they must also be informed about the potential risks of a surgical procedure and about the physical limitations of the postoperative period. In contact players, we initially consider their immediate shoulder requirements, and if they are professional or semi-professional players, we also consider the stage of the season in which they are involved. The indication for surgical treatment in this group of patients must always take the performance expectations of the athlete for the rest of the season into consideration.

### Timing for surgery

Weinstein et al. described the time point distinguishing acute versus delayed surgery as 3 weeks after the date of the shoulder injury [6]. In their comparative study, the surgical procedure was the modified Weaver–Dunn technique in 15 of 27 cases managed in the acute setting and in 14 of 17 cases managed in the chronic setting. The rest of the repairs were performed by means of AC non-absorbable sutures. Satisfactory results were obtained in 96% of cases treated in the acute phase and in 76% of cases treated in the chronic phase. The differences were statistically significant in favor of treatment in the acute phase [6].

Rolf et al. compared a group of patients treated immediately after the occurrence of shoulder injury (29 patients, using the modified Phemister technique, adding a CC fixation with sutures) with a group of patients who had undergone surgery after failure of conservative treatment (20 patients using the modified Weaver–Dunn procedure) [11]. The results were significantly superior in the group of patients managed in the acute phase [11].

Mignani et al. compared 25 patients treated in the acute phase with 15 patients treated in the chronic phase [12]. In both groups the management consisted of AC and CC temporary fixations with Kirschner wires and concomitant excision of the distal third of the clavicle. The authors reported satisfactory results in 100% of patients in the acute group and 93% of patients in the chronic group, with no statistically significant differences [12].

Dumontier et al. compared 32 patients treated in the acute phase (first 3 weeks) with 24 patients treated in the chronic phase (>3 weeks) [13]. All patients were treated by means of transposition of the coracoacromial (CA) ligament. The results were satisfactory in 81% of patients treated in the acute phase and in 79% of patients treated in the chronic phase [13]. The study reported no significant differences between groups.

Von Heideken et al. compared 22 patients treated in the acute phase (within the first 4 weeks after injury) with 15 patients treated in the chronic phase (after a minimum of 4 months of conservative measures) [14]. The technique used was ACJ fixation with a hook plate. The results were significantly superior, both in the clinical and radiological aspects, in the group of patients managed in the acute phase [14]. A summary of the main aspects of these studies is shown in Table 1.

**Table 1:** Management in the chronic setting versus management in the acute setting

Study	*n*	Type of treatment	Mean follow-up	Results
Weinstein et al. [6]	44	Modified Weaver–Dunn technique in 15/27 acute cases, and in 14/17 chronic cases. The rest of the repairs were performed by means of AC non-absorbable sutures	4 years (range 2–9)	Satisfactory results in 96% of acute cases and 76% of chronic cases. The differences were statistically significant in favor of acute cases
Rolf et al. [11]	49	29 patients using the modified Phemister technique versus a group of patients who underwent surgery after failure of conservative treatment (20 modified Weaver–Dunn)	53 months (range 20–92)	The results were significantly superior in the group of patients managed in the acute phase
von Heideken et al. [12]	37	22 patients treated in the acute phase versus 15 patients treated in the chronic phase. Hook plate in all cases	22 acute patients were re-evaluated at average of 38 months (range 15–96 months) after surgery, and 15 chronic patients were re-evaluated at an average of 36 months (range 18–62) after surgery	The results significantly favored both the clinical and radiological aspects, to the group of patients treated in the acute phase
Mignani et al. [13]	40	25 patients in the acute phase versus 15 patients in the chronic phase. In both groups the management consisted of AC and CC temporary fixations with K-wires	Unknown	Satisfactory results in 100% of patients in the acute group and 93% of patients in the chronic group. No statistically significant differences
Dumontier et al. [14]	56	32 patients in the acute phase versus 24 patients in the chronic phase. All patients were treated by means of CA ligament transposition	Acute group (mean follow-up 46 months) and chronic group (mean follow-up 51 months)	The results were satisfactory in 81% of patients treated in the acute phase and in 79% of patients treated in the chronic phase, with no significant differences

### Surgical techniques for the management of chronic ACJ instability

#### Coracoacromial ligament transposition

**Fig. 1:**
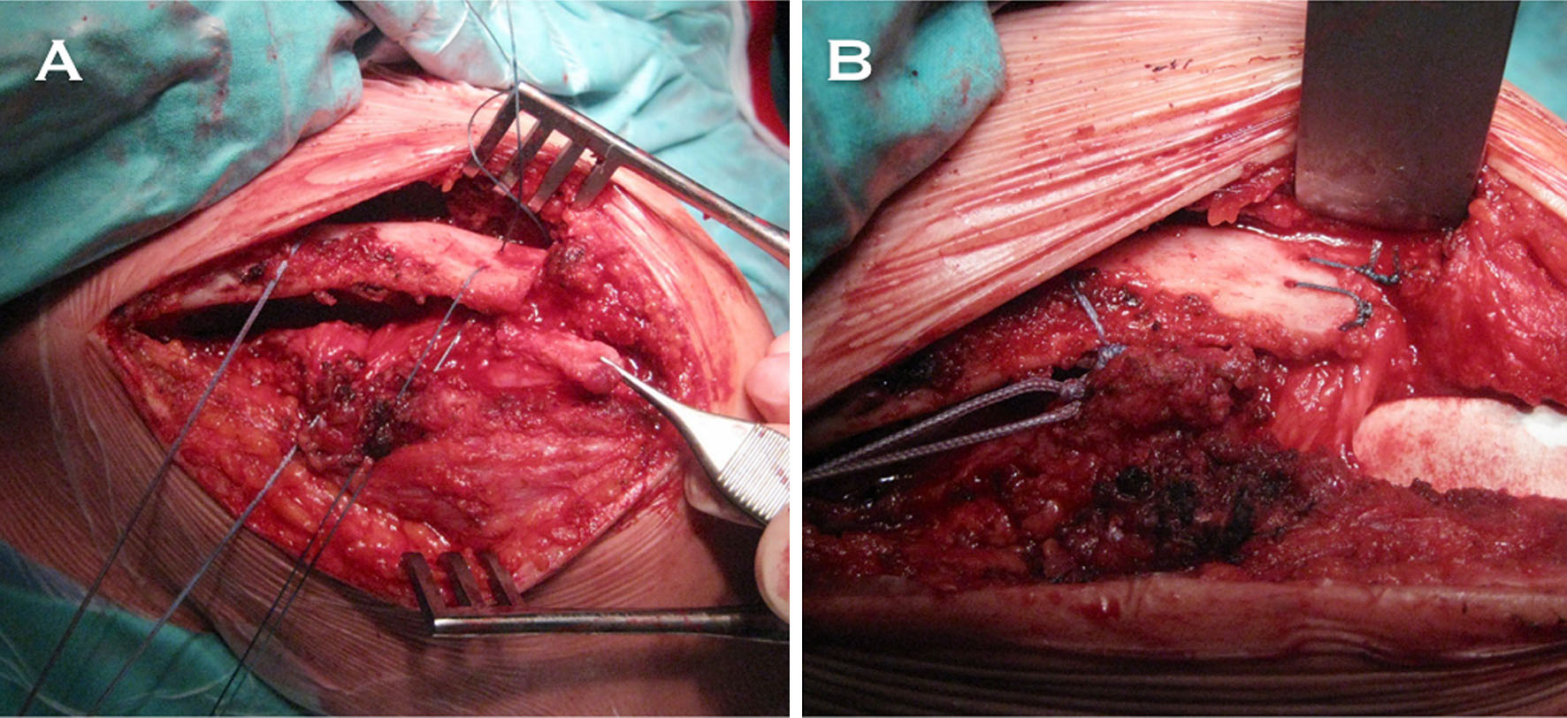
Superolateral intraoperative perspective of a left shoulder with a history of chronic ACJ dislocation, that was managed by means of a modified Weaver–Dunn procedure. **a** Visualization of the coracoacromial (CA) ligament previous to its transfer to the distal third of the clavicle. Sutures have already been passed through the bone tunnels. The most medial tunnel aimed to achieve coracoclavicular (CC) fixation. This suture was previously passed beneath the coracoid process. **b** Details of the final suture fixation. Sutures are passed through the bone tunnels created in the clavicle

The most classical method for the surgical management of chronic ACJ instability is the technique that involves transposition of the CA ligament (Fig. 1) [15, 16]. The technique described by Weaver and Dunn involves excision of the distal third of the clavicle, detachment of the AC ligament from the acromion, and transposition of this ligament to the distal third of the clavicle [16]. The modifications made to the original Weaver–Dunn procedure aimed to increase the primary mechanical stability of the fixation, by means of adding a CC fixation with subcoracoid suture loops [17], coracoid suture anchors [18], or tendon grafts. Another described modification consists of the addition of a hook plate [19].

Boileau et al. described an all-arthroscopic Weaver–Dunn–Chuinard procedure with double-button fixation for chronic ACJ dislocations [15]. The authors performed the above-mentioned technique in 10 consecutive patients with ACJ injuries (Rockwood type III or IV). After a mean follow-up of 12.8 months, the authors reported that patients were satisfied or very satisfied with the cosmesis; 9 of 10 patients returned to previous sports, and all symptoms resolved in all patients. They concluded that the bone block transfer (Weaver–Dunn–Chuinard procedure) involves the advantage of being a stronger repair, providing bone-to-bone healing by using free, autologous, vascularized tissue [15]. The authors reported that double-button fixation has the advantage of maintaining the reduction during the biological healing process. We believe that this technique involves a biomechanical disadvantage related to the transposition of the CA ligament [20].

Studies have shown the inferior characteristics of the CA ligament compared to the native ACJ [20]. The clinical outcomes obtained by means of the described modifications to the Weaver–Dunn technique have been described as satisfactory [17–19]. However, it is noteworthy that the use of the hook plate has been associated with a higher rate of complications, including infection, plate dislocation and need for re-operation [19]. Coracoid suture anchors have been associated with a higher rate of secondary displacements [18].

Two of the modifications made to the Weaver–Dunn technique have been compared (CC fixation with PDS vs hook plate) [17]. Clinical results were similar between groups, but the authors stated that the advantage of CC fixation with PDS over the hook plate relies on the fact that there is no need for a second operation for removing the implant [17].

#### Anatomical reconstruction of the CC and AC ligaments

Several biomechanical studies have demonstrated the superiority of anatomical reconstructions over other procedures with regard to the potential to emulate the properties of the native ligaments [21].

Carofino and Mazzocca developed a reconstructive technique that involves a tendon graft fixation in the native locations of the CC ligaments [22]. They performed clavicular tunnels and placed the graft in a figure-of-eight fashion, which was fixed with interferential bio-tenodesis screws [22]. The authors proposed a subcoracoid pass of the tendon graft (without coracoid tunnel), which finally rises from the coracoid to the clavicle; both ends of the graft cross between them to form the above-mentioned configuration. In a series of 106 cases with a mean follow-up of 21 months, they reported a significant improvement of the preoperative clinical results [22].

Yoo et al. described the anatomical reconstruction of the CC ligaments assisted by arthroscopy, in which three bone tunnels were performed in the native origins of the CC ligaments—two tunnels in the clavicle and one in the coracoid [23]. The authors argue that making only one tunnel in the coracoid carries a low risk of iatrogenic fracture. The described technique does not involve the use of a primary mechanical stabilizer that would protect the graft during the integration process to the bone tunnels; a reason why it can be inferred that their reconstructions may be prone to distraction forces that might affect the initially obtained ACJ reduction. In fact, although the authors report satisfactory clinical results, subtle secondary displacements were observed at final follow-up in 100% of patients in their series (13/13) [23].

In a study by Natera et al., the senior author (Dr. Sarasquete) added a CC suspension device to the anatomical reconstruction of the CC ligaments with a tendon allograft [7] in order to improve the primary mechanical fixation and thus protect the tendon graft during the integration process to the bone tunnels and reduce the rate of secondary vertical displacements, Likewise, the study group led by the above-mentioned author described the use of two suspension devices with two tunnels in the coracoid, a technique that in the acute setting would provide greater resistance to vertical translation [24]. A summary of the main aspects of the cited biomechanical studies is shown in Table 2.

**Table 2:** Summary of the main aspects of the cited biomechanical studies

Study	Purpose	Treatment methods	Results	Conclusion
Lee et al. [3]	To compare biomechanical properties of native CC ligaments versus tendon graft reconstructions versus other methods	11 human cadaveric shoulders were tested to failure to compare the biomechanical properties of the native CC ligaments, CA ligament transfer, Mersilene suture repair, Mersilene tape repair, and tendon graft reconstructions with gracilis, semitendinosus, and long toe extensor	Reconstructions with semitendinosus, gracilis, or long toe extensor tendon grafts had superior initial biomechanical properties compared with CA ligament transfer; failure strengths were as strong as those of the native CC ligaments	Tendon graft reconstruction may be an alternative to CA ligament transfer and may provide a permanent biologic reconstruction with superior initial biomechanical properties
Michlitsch et al. [16]	To compare the biomechanical characteristics of a modified Weaver–Dunn reconstruction and an ACJ reconstruction with free-tissue graft for reconstruction of both CC and AC ligaments	6 pairs of cadaveric shoulders had a modified Weaver–Dunn reconstruction on 1 side and the contralateral side had a graft reconstruction of CC and AC ligaments. Load-to-failure was performed	AP and superior-inferior (SI) translation of the ACJ reconstruction was significantly less than that of the modified Weaver–Dunn under all loading conditions	ACJ reconstruction with free-tissue graft for both CC and AC ligaments demonstrates initial stability significantly better than a modified Weaver–Dunn and similar to that of intact specimens
Grutter et al. [17]	To compare the modified Weaver–Dunn procedure, the anatomical AC reconstruction using palmaris longus graft, and anatomical AC reconstruction using flexor carpi radialis graft	The native ACJ in 6 fresh-frozen cadaveric upper extremities was stressed to failure under tension in the coronal plane. Each repair was stressed to failure	Load to failure for native ACJ complex was 815 N, modified Weaver–Dunn 483 N, anatomical AC reconstruction with palmaris longus 326 N, and anatomical AC reconstruction with flexor carpi radialis 774 N	Anatomical AC reconstruction with a flexor carpi radialis tendon graft re-creates the tensile strength of the native ACJ complex and is superior to a modified Weaver–Dunn repair
Dawson et al. [20]	To compare the stability of the ACJ and biomechanical characteristics of the ACJ capsule and CC ligaments	AP and SI ACJ translations were quantified in 6 cadaver matched pairs. Either the ACJ capsule or CC ligaments were transected, and measurements were repeated. The biomechanics of the remaining ACJ capsule or CC ligaments were compared	Significant increases in AP translation with the cut ACJ capsule, and significant increases in SI translation with the cut CC ligaments	The ACJ capsule contributes significantly to the ACJ stability, especially in the AP plane
Deshmukh et al. [30]	To determine biomechanical basis for augmenting the Weaver–Dunn with supplemental fixation	Native ACJ motion was measured. AC and CC ligaments were cut, and 1 of 6 reconstructions was performed: Weaver–Dunn, suture cerclage, and 4 different suture anchors. ACJ motion was reassessed, cyclic loading test was performed, and failure load was recorded	Weaver–Dunn reconstructions failed at a lower load. Reconstruction using augmentative fixation allowed less AC motion than Weaver–Dunn reconstruction, but more motion than the native ligaments	Although none of the augmentative methods tested restored ACJ stability to normal, all proved superior to the Weaver–Dunn reconstruction alone.
Abat et al. [33]	To evaluate the vertical biomechanical behavior of two techniques for the anatomical repair of the CC ligaments	18 human cadaveric shoulders. 3 groups were formed–group I, control; group II, double tunnel in clavicle and 1 in coracoid (with two CC suspension devices); group III, repair in ‘V’ configuration with two tunnels in clavicle and one in coracoid (with one CC suspension device). The force required for failure was analyzed	Comparison of the three groups did not find any significant difference despite the loss of resistance presented by group III	Anatomical repair of CC ligaments with a double system (double tunnel in the clavicle and in the coracoid) permits vertical translation that is more like that of the ACJ

#### Synthetic grafts

The use of synthetic ligament reconstructions is an option that could be considered for the treatment of chronic ACJ instability. The synthetic grafts most commonly used are the Ligament Advanced Reinforcement System (LARS^®^; Surgical Implants and Devices, Arc-sur-Tille, France), the Dacron^®^ graft and the Ligastic^®^ [25, 26]. Several authors reported satisfactory clinical results with the LARS^®^ [34], and unsatisfactory results with the Dacron^®^ [25] and the Ligastic^®^ [26]. With regard to the Dacron^®^ vascular prostheses, Fraschini et al. reported a complication rate of 43.3% (13/30 patients), in which 23.3% (7/30 patients) had a graft tear [25]. Regarding the LARS^®^, the rate of graft tears described by the authors was 3.3% (1/30 patients) [25].

Regarding the Ligastic^®^, Mares et al. described a rate of clavicular osteolysis of 22% (6/27 patients) [26]. In fact, these authors reported in their study that they are currently rejecting the use of this type of implant, and advising against its use. However, further studies are needed to clarify the role of synthetic grafts in the management of chronic ACJ injuries.

Muccioli et al. compared the outcomes of ACJ reconstruction with the LARS^®^ in professional athletes with non-professional athletes at a 2-year minimum follow-up. They found that all clinical (Oxford and Constant) scores, as well as patient satisfaction, improved significantly from preoperative to follow-up intervals, with no differences between groups, and only 2% of failures (re-dislocations) [27]. On the other hand, Fauci et al. compared the clinical and radiographic outcomes of ACJ stabilization performed in patients with chronic ACJ dislocation using a biological allograft or a synthetic ligament, and reported that the ʽbiological’ group achieved significantly better clinical scores than the 'synthetic' group, at both 1- and 4-year follow-up. The authors concluded that the biological graft afforded better clinical and radiographic outcomes than the synthetic ligament in patients with chronic ACJ instability [28].

#### Dynamic stabilization of the ACJ

An osteotomy is made to the coracoid process, which is later transferred to the inferior aspect of the clavicle with the attached conjoined tendon [29]. The bone block is fixed to the clavicle by means of a screw with a spike washer. In this way, the conjoined tendon is converted to a depressor of the clavicle. This concept does not directly address the pathomechanics of an ACJ injury in which, rather than a superior displacement of the clavicle, it is the scapula that descends [1]. Despite this issue, the technique has been used in both the acute and chronic settings with satisfactory results [30].

#### Distal third clavicle excision

Excision of the distal third of the clavicle (Mumford procedure) may represent a solution to a painful chronic ACJ injury (grade I–III) [31]. Osteoarthritic changes have been described to be mostly restricted to type I and type II injuries, since the greater separation of the bone ends in higher-grade injuries may prevent the development of this complication [31]. However, degenerative changes in the articular disc and lateral end of the clavicle may be found during surgery and might be a source of pain in high-grade injuries. This technique must involve the resection of only 5 mm of the distal third of the clavicle, since (in cases of ACJ injuries grade I–II) the trapezoid ligament is only 2.5 cm medial to the distal end of the clavicle [4]; more generous resections could affect the clavicular insertion of the trapezoid ligament.

#### Authors preferred technique

This technique has been previously described [7].

**Fig. 2:**
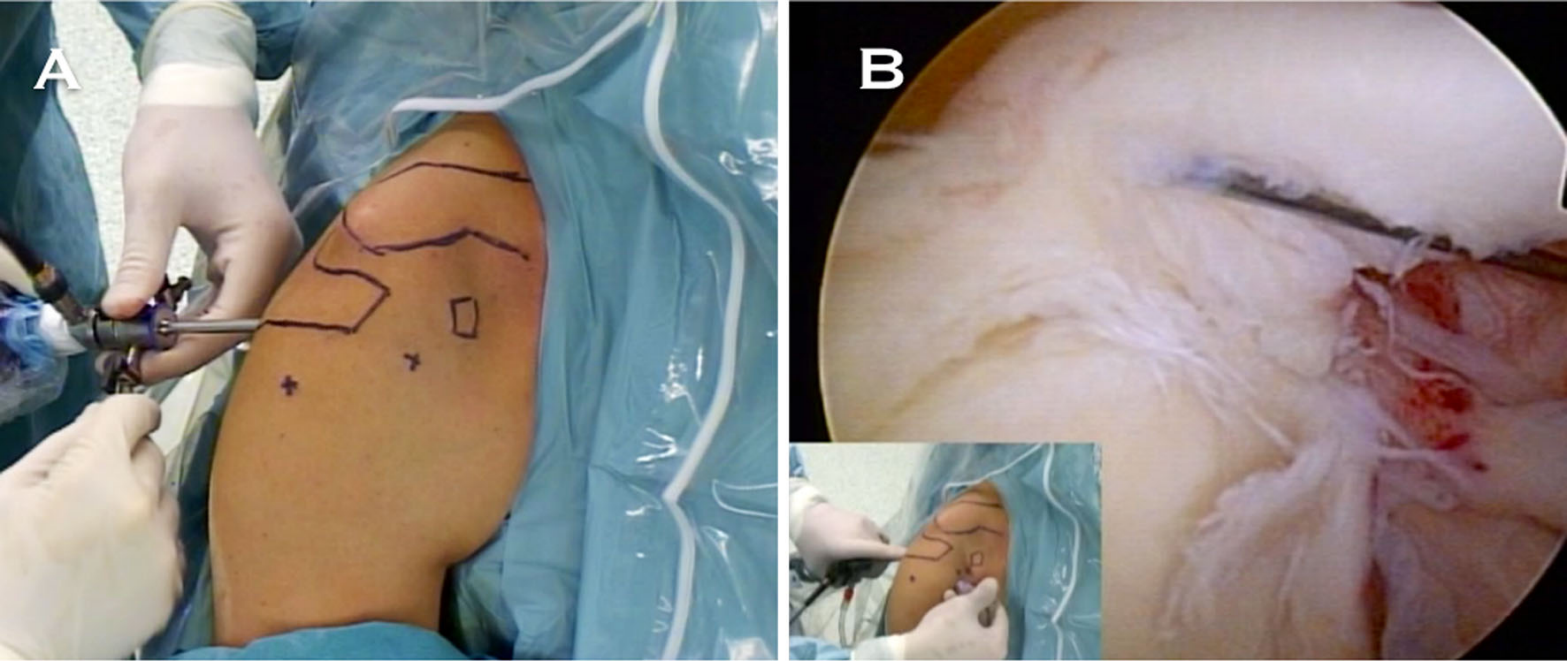
**a** Anterolateral perspective of a right shoulder positioned in the operating room, with a history of a chronic grade V ACJ injury. **b** Biceps-labrum complex viewed from the posterior portal. Notice the degenerative aspect of the biceps insertion, which indicates an associated glenohumeral injury

**Fig. 3:**
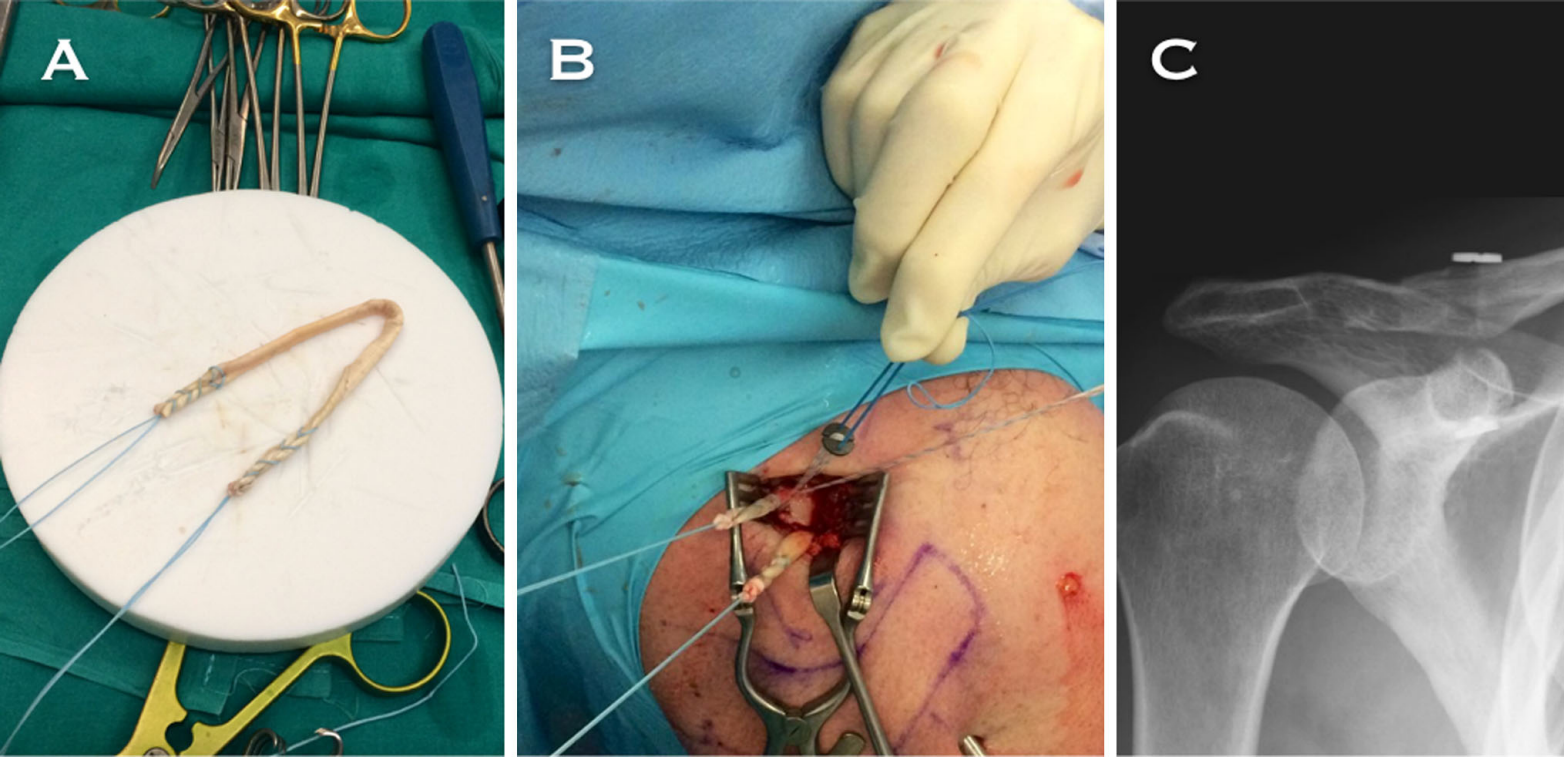
**a** Semitendinous allograft after being sutured with a metal-core suture in both of its limbs. **b** Both limbs of the graft coming out of the clavicle once fixed in both tunnels with bio-tenodesis interference screws. The ZipTight is tied by threading the sliding suture in the washer. **c** AP X-ray of a right shoulder in which an anatomical reconstruction of CC ligaments with tendon allograft was performed in the chronic setting. Observe the trapezoid tunnel in the clavicle, lateral to the conoid tunnel in the clavicle, through which also passes the suspension device

We perform an arthroscopy-assisted reconstruction in order to be able to diagnose and treat possible associated glenohumeral injuries (Fig. 2). We propose anatomical reconstruction of the CC ligaments using a semitendinosus tendon allograft (Fig. 3a, b). In Fig. 3c, the radiological aspect of a right shoulder in which this technique was performed can be appreciated. In a contact player, we prefer to use a tendon autograft, which may be the ipsilateral palmaris longus.

The technique implies one tunnel at the coracoid, and two tunnels at the clavicle. These tunnels aim to emulate the anatomical locations of the CC ligaments. We also add a CC suspension device in order to guarantee primary stability of the reconstruction.

A subacromial approach to the base of the coracoid is performed in association with a Mumford procedure. A transverse skin incision over the clavicle is performed. The conoid native insertion is 4.5 cm medial to the distal end of the clavicle and the trapezoid native insertion is 2.5 cm and subtly anterior when compared to the conoid [4].

**Fig. 4:**
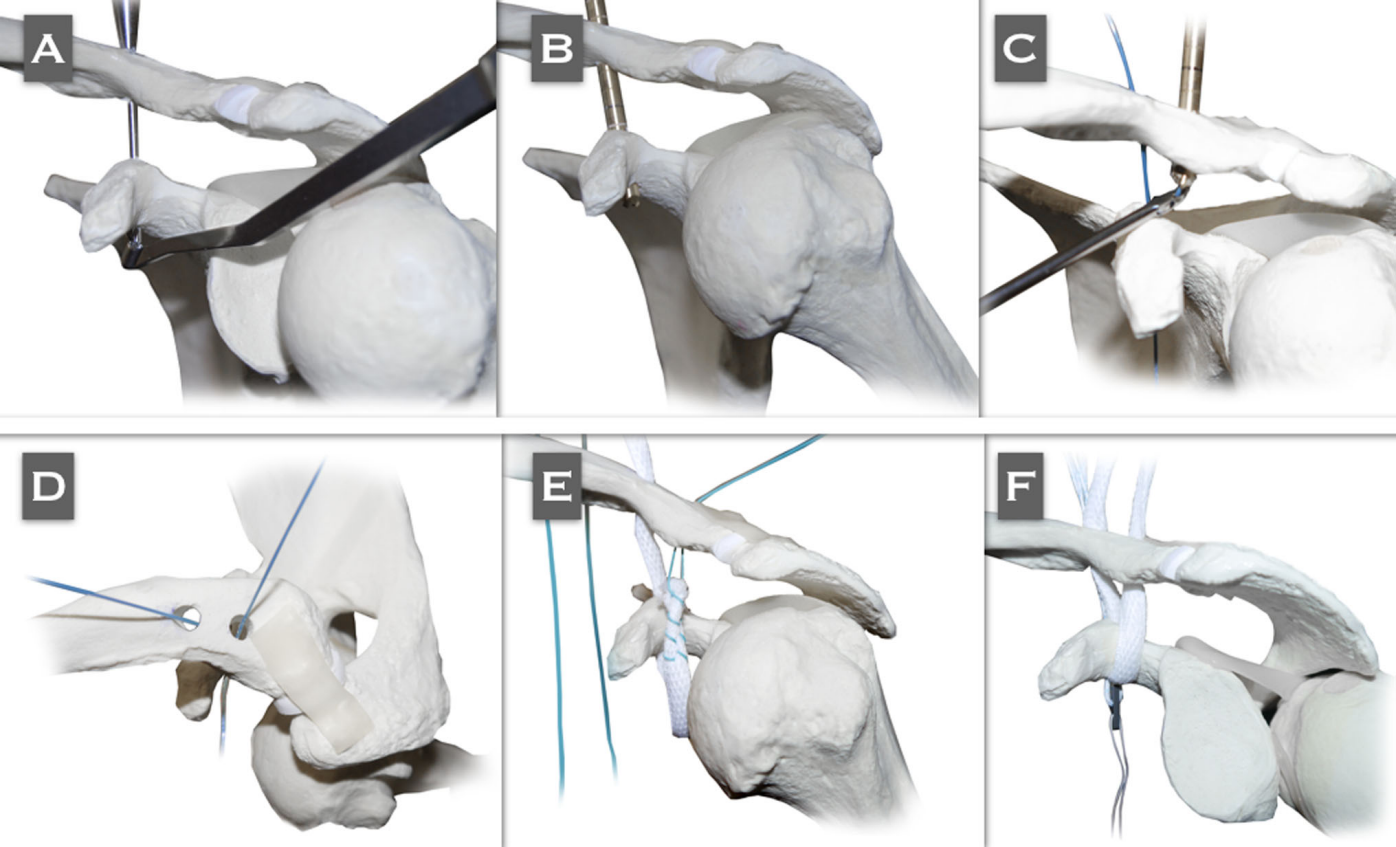
Reproduced with permission and copyright© of Arthroscopy Techniques, Elsevier. **a** The AC drilling guide is placed at the coracoid base with the sliding tube of the guide in the superior aspect of the clavicle, 4.5 cm medial to its lateral border (conoid native origin). A 2.4-mm K-wire is passed through the AC guide. **b** A cannulated 4.5- to 6-mm (depending on the graft diameter) drill is passed over the K-wire and comes out from the inferior aspect of the coracoid. **c** A shuttle 1-mm PDS suture is passed through the cannulated drill located in the trapezoid tunnel. The PDS is recovered with a grasper from the anterior portal. **d** Superior perspective of the clavicle in which both shuttle sutures are emerging from the tunnels. **e** The PDS that arises from the trapezoid tunnel in the clavicle is pulled out in a cranial direction to recover the limb of the graft that is going to surround the base of the coracoid at its lateral aspect, coming from its tunnel and then being directed laterally and superiorly, configuring the anatomical 'V' shape of the graft. **f** Once the graft has passed through both clavicle tunnels, the ZipTight is tied to the distal limb of the shuttle FiberWire that is still free in the conoid tunnel

A cross section of the deltotrapezial fascia is performed, and the AC drilling guide is placed at the base of the coracoid with the sliding tube at the superior aspect of the clavicle, 4.5 cm medial to its distal end (conoid native origin) (Fig. 4a). A K-wire is passed followed by the cannulated drill. The K-wire is removed and the cannulated drill is maintained in the same position (Fig. 4b). Subsequently, the same procedure should be performed for the clavicular tunnel of the trapezoid ligament. Shuttle sutures are passed through the cannulated drills (Fig. 4c). Two metal-core sutures are tied to the distal end of the shuttle suture that passes through the coracoid. A superior perspective of the clavicle shows both shuttle sutures emerging from the tunnels (Fig. 4d).

One of the metal-core sutures passing through the conoid tunnel is temporarily tied to one of the ends of the tendon graft. The other end of the graft is temporarily tied to the shuttle suture, which is coming out of the trapezoid clavicle and exits through the anterior portal.

The graft is passed by means of pulling cranially on the metal-core suture that comes out of the conoid tunnel. Subsequently, the shuttle suture which is coming out of the trapezoid clavicle tunnel is pulled in a superior direction; the graft is directed laterally and superiorly, conforming to the anatomical 'V' configuration of the reconstruction (Fig. 4e).

One of the ends of the shuttle metal-core suture is still free in the conoid tunnel. This suture is now tied to the CC suspension device, and pulled out in a cranial direction so the device passes in a retrograde direction (Fig. 4f).

**Fig. 5:**
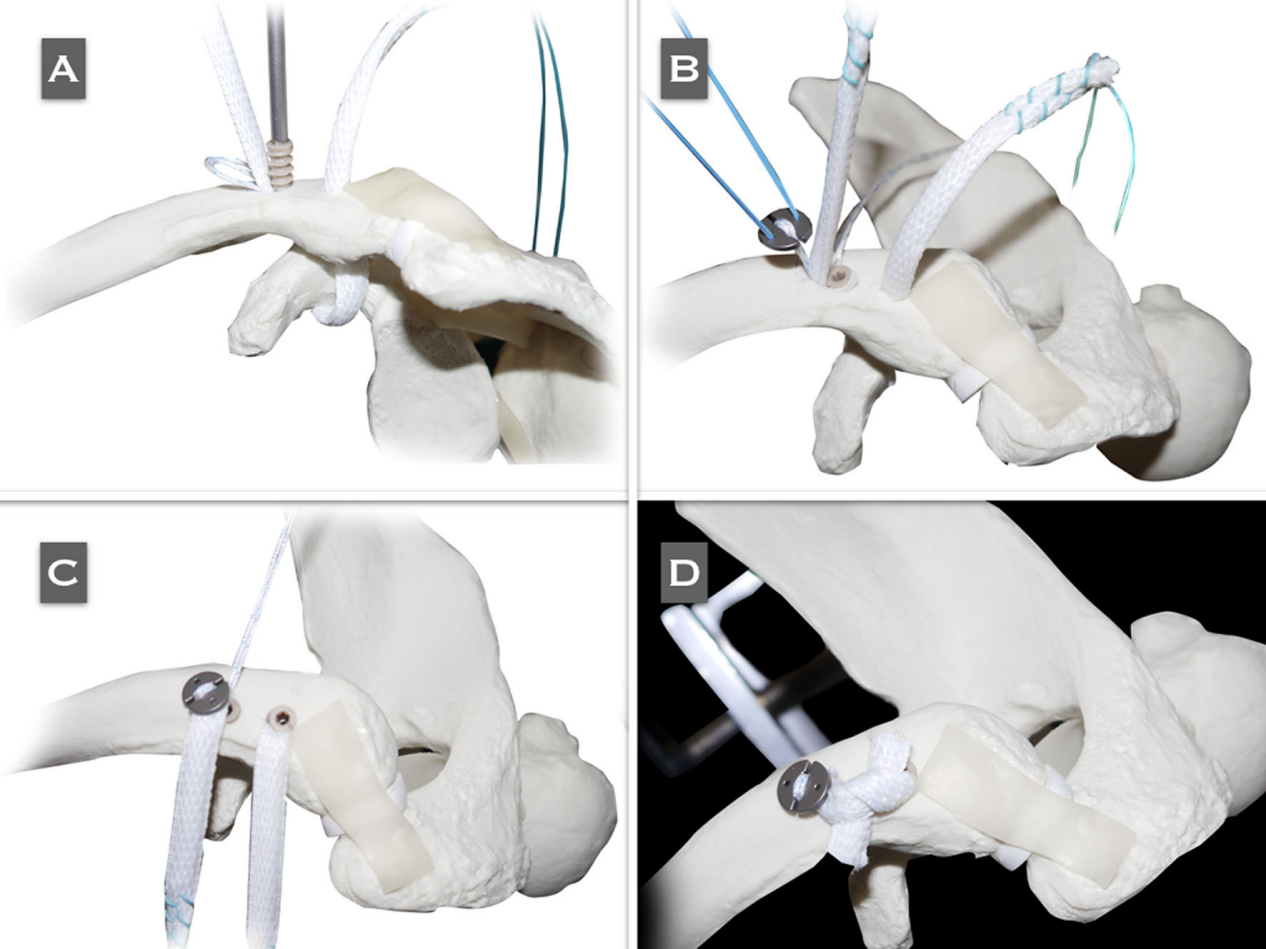
**a** Before the ZipTight is tensioned, the graft should be fixed in the clavicular portion of the conoid tunnel with a 4.5- to 5.5-mm (same diameter of the tunnel) bio-tenodesis interference screw. Reproduced with permission and copyright© of Arthroscopy Techniques, Elsevier. **b** Both limbs of the graft coming out of the clavicle when fixed in both tunnels with bio-tenodesis interference screws. The ZipTight is tied by threading the sliding suture in the washer. To avoid any harm to the sutures of the ZipTight with the screw, the graft should be placed in an intermediate position between the screw and the sutures. **c** The ZipTight has been tied by pulling alternatively on both limbs of the blue traction sutures in a cranial direction to make the washer go down until it touches the clavicle and self-locks, providing mechanical stabilization of the reconstruction. **d** Both limbs of the graft are crossed over each other and sutured to themselves. The remnant of the graft is sectioned and removed

The graft is fixed in the clavicular portion of the tunnels with bio-tenodesis interferential screws (Fig. 5a). The washer should be threaded with the sliding sutures, in order to be able to bring it down until it is applied over the clavicle (Fig. 5b). The assistants must reduce the ACJ by pushing the elbow upwards and the clavicle downwards at the same time. The CC suspension device is now locked (Fig. 5c). Both limbs of the graft are crossed over each other and sutured to themselves (Fig. 5d). The remaining graft is sectioned and removed. The deltotrapezial fascia is carefully reconstructed.

**Fig. 6:**
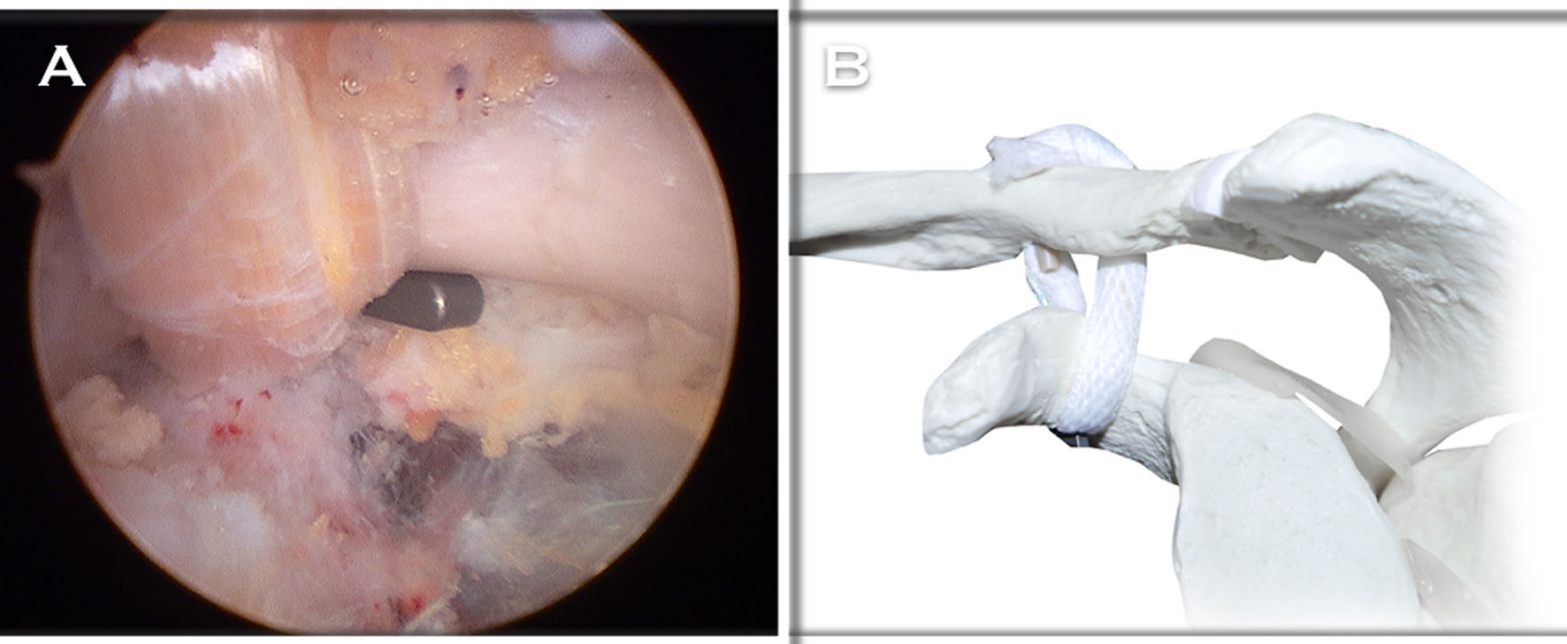
**a** Final arthroscopic view from the lateral portal. The graft is coming out of the coracoid tunnel, ascending toward the trapezoid tunnel in the clavicle. The flip of the ZipTight is supported in the inferior aspect of the coracoid. **b** Final anatomical 'V' configuration of the CC reconstruction, with the flip of the ZipTight supported in the inferior aspect of the coracoid and both limbs of the graft are crossed over each other and sutured to themselves. Reproduced with permission and copyright© of Arthroscopy Techniques, Elsevier

The described technique provides the advantages of minimally invasive surgery, avoids the biomechanical disadvantages related to rigid metal hardware procedures, offers greater biomechanical resistance thus minimizing the risk of secondary displacements related to non-anatomical techniques, and combines primary mechanical stabilization and definitive biological stabilization represented by the graft, once integrated to the bone (Fig. 6a, b).

The results obtained with this technique have been published previously [32]. Ten patients with a mean age of 41 years underwent surgery after failure of conservative measures. The clinical outcomes showed a significant improvement from the visit prior to surgery to the last follow-up in all patients, and no secondary vertical instability was registered in any of the cases [32].

#### Fixation method of the tendinous allograft in the coracoid process

It has been reported that suture subcoracoid loops tend to dislocate anteriorly due to the ascending slope that is represented by the most caudal portion of the base of the coracoid [33]. It has also been shown that the use of subcoracoid suture loops can involve a shear deleterious effect on the bone [34].

Other authors propose techniques that do not involve making tunnels at the coracoid, but pass the graft around the caudal portion of the bone. We think that by taking into consideration the fact that there is no contact between the cancellous bone and the collagen of the tendon graft [22], integration of the graft might not be developed.

## Postoperative management

Regardless of the chosen technique, due to the fact that biological augmentation should be employed in the chronic setting, there should be a protection period of the reconstruction in order to guarantee integration of the graft to the bone tunnels [7].

The shoulder should be maintained in a sling for 46 weeks. Patients should be allowed from the beginning to fully and actively move the elbow, wrist, and hand and should be allowed to passively move the shoulder into no more than 90° of elevation in the plane of the scapula. The exercise program should be started after the sixth week. Pendulum exercises must begin in the fourth week, and active range of motion is allowed from the sixth week onwards. Exercises to regain strength are initiated once the patient achieves full, pain-free passive and active range of motion. These exercises are primarily directed toward scapular stabilization. Return to work without restrictions is allowed at 12–16 weeks after surgery, and contact sports, as well as tasks requiring major efforts should be avoided for 4–6 months after surgery. The achievement of a full recovery and the return to maximum strength and function can take from 9−12 months.

## Complications

The profile of complications that can be expected after surgery for ACJ instability depends on whether the reconstruction is performed in the acute or chronic setting, on the type of fixation used, and on whether the reconstruction is performed using arthroscopy-assisted or open surgery. The rate of complications according to different studies is shown in Table 3.

**Table 3:** Rate of complications according to different studies

Study	*n*	Technique	Mean follow-up (months)	Rate of complications	Type of complications
Tauber et al. [18]	24	12 patients, modified Weaver–Dunn 12 patients, autogenous semitendinosus tendon graft	37	12.5% (3/24)	Semitendinous group, 1 mild loss of reduction. 1 mild hyperesthesia of the saphenous nerve. Weaver–Dunn group, 1 superficial wound infection
Boileau et al. [25]	10	All-arthroscopic Weaver–Dunn–Chuinard procedure with double-button fixation	12.8	20% (2/10)	1 Superficial infection of the superior portal. 1 lateral migration of the subcoracoid EndoButton
Carofino et al. [31]	22 reconstructions in 21 patients. 16 were available for follow-up	Open anatomical CC ligament reconstruction	21	18.75% (3/16)	1 Persistent ACJ pain. 1 chronic infection, requiring removal of the allograft and latissimus flap coverage. 1 loss of reduction
Yoo et al. [32]	13	Arthroscopically assisted anatomical CC reconstruction with tendon graft	17	23% (3/13)	3 Loss of reduction. In all patients, mild displacement
Fraschini et al. [34]	60 managed surgically and 30 managed conservatively	30 CC reconstructions with DACRON^®^, 30 CC reconstructions with LARS^®^	15	43% (13/30) in the DACRON^®^ group and 3.3% (1/30) in the LARS^®^ group	DACRON^®^ group: 7 recurrences due to neoligament rupture, 4 aseptic separations, 1 clavicle fracture and 1 coracoid fracture. LARS^®^ group: 1 neoligament rupture
Cook et al. [43]	10	Arthroscopic CC ligament reconstruction with GraftRope (Arthrex) plus tendon allograft	9.7	80% (8/10)	8 Loss of reduction, 4 revision surgeries

With regard to infection rates, a systematic review of the literature reports that the overall rate of superficial infections is approximately 3.8% for arthroscopic procedures [35], in contrast to a rate of up to 5% for procedures performed by open surgery [35], and up to 8% in those procedures in which a tendon graft was used [36, 37].

The failure rate after fixation in the chronic setting using only a tendon graft, has been reported to be approximately ≥50% [35, 38], while the failure rate after management in the acute setting has been reported to be approximately 26.8% [35].

It has been reported that these differences may be due to the fact that the tendon graft tends to lengthen over time, and it may also emulate a 'windshield' effect at the level of the clavicular tunnels, a situation that eventually ends with widening of the tunnels [39].

Regarding the incidence of fractures of the coracoid process, it has been reported that the overall rate (both mono-tunnel and double-tunnel techniques) is approximately 5.3% [35].

## Non-surgical management of chronic ACJ instability

**Fig. 7:**
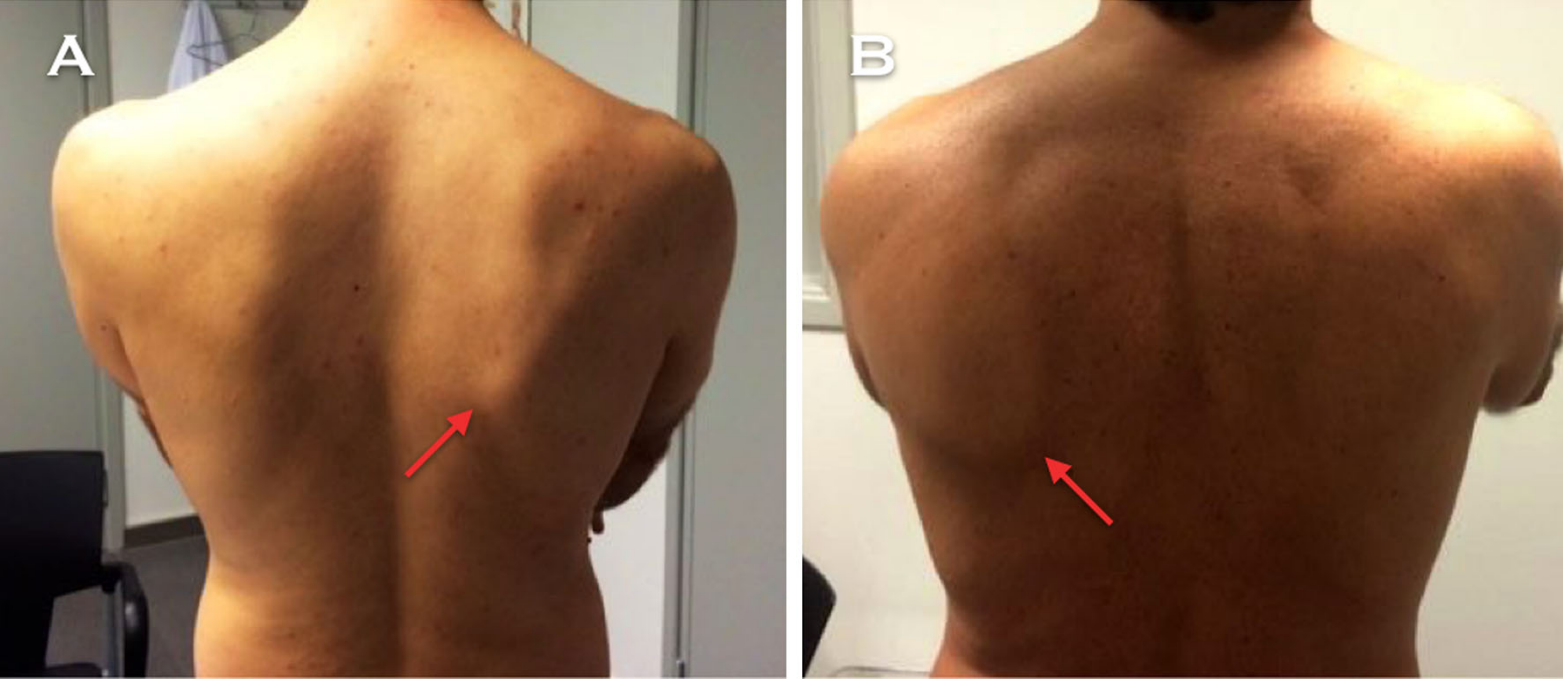
**a** and **b** Posterior perspective of two patients performing shoulder forward flexion. Notice that the inferomedial border of the right scapula (*red arrows*) shows a prominence. These two patients had a history of chronic unstable ACJ injuries that were conservatively treated

Gumina et al. reported that the prevalence of scapular dyskinesis (Fig. 7) in patients with chronic ACJ instability (Rockwood grade III) can be up to 70.6% [40], and that the prevalence of SICK scapula [41] (Scapular malposition, Inferior medial border prominence, Coracoid pain and malposition, and dyskinesis of scapular movement) can be up to 58.3% [40]. This group of patients might develop persistent shoulder pain that could make them unable to return to their previous daily life activities [42]. The occurrence of modifications in the scapular orientation leads to cinematic alterations of the muscles, thus perturbing the shoulder girdle biomechanics. Likewise, it has been shown that the prevalence of scapular dyskinesis in those patients managed surgically is lower when compared to patients managed non-surgically [2, 40].

Patients with this syndrome may refer shoulder pain at the ACJ and at the coracoid, posterior shoulder pain sometimes irradiated to the cervical paraspinal region and to the lateral aspect of the arm, or even radicular symptoms.

Carbone et al. proposed a rehabilitation protocol for patients with scapular dyskinesis [43]. The protocol consists of 12 exercises aimed to strength the scapular muscles. These authors described a series of 24 patients with a history of chronic ACJ instability (grade III) in which 100% (24/24) had scapular dyskinesis and 58.33% (14/24) had SICK scapula [43]. Twelve months after having accomplished the rehabilitation protocol, 21.73% (5/23) of the patients still had scapular dyskinesis and 17.4% (4/23) still had SICK scapula. They concluded that scapular dyskinesis and SICK scapula secondary to chronic ACJ instability might show improvement within 6 weeks of starting this rehabilitation protocol.

## Discussion

### Arthroscopy-assisted surgery versus open surgery

With regard to the advantages that arthroscopy-assisted surgery may offer over open surgery in cases of chronic ACJ instability, it is important to mention that associated glenohumeral lesions can be diagnosed and treated [7]. Some authors have reported that the incidence of lesions associated with unstable ACJ injuries can be up to 30% [44]. In the management of chronic ACJ instability, it is important to guarantee that there is no interposition of the deltotrapezial fascia between the clavicle and the acromion, a situation that can only be accomplished by means of making a mini-approach just above the ACJ. Once anatomical reduction of the ACJ has been reached, the deltotrapezial fascia should be carefully reconstructed in order to ensure adequate vertical and horizontal stability [7].

### Anatomical versus non-anatomical reconstructions

Anatomical AC and CC ligament reconstruction techniques have become increasingly popular. Several clinical and biomechanical studies have shown their superiority in reproducing the strength and stiffness of the native ACJ complex when compared to other reconstructive techniques [20, 36, 45]. Biomechanical studies of ACJ reconstructions with free-tissue grafts for both the CC and the AC ligamentous complex have shown that these techniques provide ACJ stability similar to that of the native ACJ [45]. Likewise, it is currently clear that by taking into consideration the biomechanics and the resistance of the reconstruction that anatomical procedures are superior techniques when compared to the classical Weaver–Dunn technique [45].

Lafosse et al. describe an arthroscopic technique indicated for cases of chronic ACJ instability, in which they propose CA ligament transfer in order to reproduce the function of the torn CC ligaments [8]. It has been reported that transposition of the CA ligament of the Weaver–Dunn technique offers a lower resistance to vertical translation than anatomical CC reconstructions with tendon grafts [20].

LaPrade et al. described an open non-anatomical technique in which they propose the use of a semitendinosus allograft, which passes through a tunnel in the clavicle and another in the coracoid [9]. This technique entails a biomechanical disadvantage that does not take into account the anatomical location of the CC ligaments [9]. The authors recognize that in some patients, an elongation of the graft may be developed, a situation that may result in persistent ACJ instability in the vertical plane [9].

In a prospective, comparative, clinical study, Tauber et al. showed that anatomical ligament reconstruction of the conoid and trapezoid ligaments with tendon grafts results in superior outcomes compared to the modified Weaver−Dunn technique [36].

### Anteroposterior (AP) stabilization

The shoulder community has shown an increasing interest in anatomical CC ligament reconstruction, because these concepts aim to recreate the force vectors of both the conoid and trapezoid ligaments, thus restoring both horizontal and vertical instability. Despite the recent development of numerous reconstructive techniques, residual AP post-surgical instability remains a matter of concern [46]. Likewise, the importance of simultaneous reconstruction of the AC ligaments has been widely studied and demonstrated [47]. It has been reported that patients who underwent surgery for unstable ACJ injury, and show remaining AP post-surgical instability, may have significantly inferior clinical results [48]. Likewise, it has been also reported that persistent AP post-surgical instability is the only factor that may adversely affect the clinical outcomes [48]. For this reason, reconstructive strategies must give the same importance to AC reconstruction as to CC reconstruction [49].

### Arthroscopic approach to the coracoid process

Some authors propose a direct skin incision over the tip of the coracoid, blunt dissection and identification of its base, in order to place the drilling guide [50]. These techniques are performed in a 'blind' manner, and therefore lack the precision that direct visualization may provide. To guarantee a proper view of the lower portion of the base of the coracoid, several arthroscopic techniques that facilitate the process of tunnel-making and implant-positioning have been described [7–9]. Glenohumeral access involves the need to release the superior and middle glenohumeral ligaments, in order to gain access to the coracoid process [51]. On the other hand, subacromial access to the coracoid has the advantage over glenohumeral access, as it does not involve the potential deleterious effect that may result from the release of the superior and middle glenohumeral ligaments [7].

## Overview

Considering all the procedures described in this review, patients with shoulder symptoms resulting from chronic ACJ instability may benefit from surgical treatment. The procedures considered for the management of chronic ACJ instability should take into account the biological aspects; for this reason the use of either a tendon graft, ligament or osteotendinous transposition should always be considered. Likewise, the fundamental role that primary mechanical fixation may play should to be taken into account, in order to protect the integration period of biological augmentation to the bone.
